# Olfactory bulb granule cells: specialized to link coactive glomerular columns for percept generation and discrimination of odors

**DOI:** 10.1007/s00441-020-03402-7

**Published:** 2021-01-06

**Authors:** Veronica Egger, Thomas Kuner

**Affiliations:** 1 Institute of Zoology, Regensburg University, Universitätsstr. 30, 93040 Regensburg, Germany; 2 Department of Functional Neuroanatomy, Institute for Anatomy and Cell Biology, Heidelberg University, Im Neuenheimer Feld 307, 69120 Heidelberg, Germany

**Keywords:** GABA, Granule cells, Olfactory processing

## Abstract

The role of granule cells in olfactory processing is surrounded by several enigmatic observations, such as the purpose of reciprocal spines and the mechanisms for GABA release, the apparently low firing activity and recurrent inhibitory drive of granule cells, the missing proof for functional reciprocal connectivity, and the apparently negligible contribution to lateral inhibition. Here, we summarize recent results with regard to both the mechanisms of GABA release and the behavioral relevance of granule cell activity during odor discrimination. We outline a novel hypothesis that has the potential to resolve most of these enigmas and allows further predictions on the function of granule cells in odor processing. Briefly, recent findings imply that GABA release from the reciprocal spine requires a local spine action potential and the cooperative action of NMDA receptors and high voltage-activated Ca^2+^ channels. Thus, lateral inhibition is conditional on activity in the principal neurons connected to a granule cell and tightly intertwined with recurrent inhibition. This notion allows us to infer that lateral inhibition between principal neurons occurs “on demand,” i.e., selectively on coactive mitral and tufted cells, and thus can provide directed, dynamically switched lateral inhibition in a sensory system with 1000 input channels organized in glomerular columns. The mechanistic underpinnings of this hypothesis concur with findings from odor discrimination behavior in mice with synaptic proteins deleted in granule cells. In summary, our hypothesis explains the unusual microcircuit of the granule cell reciprocal spine as a means of olfactory combinatorial coding.

## Introduction

The role of granule cells in olfactory processing is surrounded by several enigmatic findings (Shepherd et al. 2007; Burton 2017). Here, we present recent results and outline a novel hypothesis that has the potential to resolve most of these enigmas and allows further predictions on the function of granule cells in odor processing. Our focus is the synaptic interaction of mitral cells and granule cells via the reciprocal synapse, the mechanisms of signal processing in granule cells with an emphasis on their spines/gemmules, and the network consequences of these functions and mechanisms, especially with regard to odor discrimination. Plasticity at this synapse, adult neurogenesis of granule cells and higher order processing beyond the olfactory bulb are not within the scope of our review.

As described in previous chapters (*cross references to be added in proof*) in more detail, odorant molecules bind to olfactory receptor neurons in the nasal epithelium. The axons of these receptor neurons terminate in glomeruli of the olfactory bulb (OB) where they interact with local interneurons and the apical dendritic tufts of the bulbar projection neurons, the mitral and tufted cells (MTC), which in turn send their axons mostly to olfactory cortical areas (Igarashi et al. 2012; Nagayama et al. 2014). A single glomerulus usually receives convergent input from olfactory sensory neurons that all express the very same olfactory receptor (Belluscio et al. 2002). Activation of a given olfactory receptor will in turn activate its associated glomerular unit and the neurons belonging to the respective glomerular column. This unit could be considered an input channel that carries and further processes information as defined by the properties of the olfactory receptor generating the initial signal. Any given olfactory receptor is usually sensitive to a set of similar molecular structural elements with varying affinity (Malnic et al. 1999). Thus, even a single odorant can activate multiple glomerular units. Since the odor space consists of an enormous, nearly infinite number of possible odorants, olfactory processing needs to provide extensive inherent flexibility, which is in part covered by combinatorial coding: Odors are encoded as specific combinations of wide-spread glomerular activities. Due to the large number of olfactory receptors (~ 1000 in rodents (Mombaerts 1999)), this combinatorial principle allows to accommodate vast numbers of different stimuli. However, the problem arises of how to synthesize an “odor image” out of this highly distributed activity. This synthesis has been suggested to happen downstream of the bulb, in pyramidal cells of the olfactory cortex that integrate inputs from active MTCs of the entire bulb (Franks and Isaacson 2006; Apicella et al. 2010; Ghosh et al. 2011; Igarashi et al. 2012).

Inhibition both within and across glomerular columns is a key element of OB computations, as reflected in the extraordinarily large fraction of inhibitory interneurons. In the OB, the ratio of the inhibitory versus the excitatory neuron numbers amounts to roughly 80% versus 20% of the total population, which is the inverse of ratios in vertebrate cortex and hippocampus (Shepherd et al. 2004). The inhibitory interactions take place within a two-stage network (Aungst et al. 2003) of local and long-range connections, (1) within the glomerular layer, where a highly diverse set of inhibitory neurons forms synapses with the terminals of the receptor neurons and the apical dendritic tufts of MTCs and (2) in the external plexiform layer (EPL), where the long lateral MTC dendrites engage in reciprocal dendrodendritic interactions with granule cells (GC) and other inhibitory neurons, most prominently parvalbumin (PV+) neurons. These interactions are the subject of several excellent recent detailed reviews (Nagayama et al. 2014; Burton 2017) and represent a fundamental processing step in forming the odor representation on the level of the OB.

**Fig. 1:**
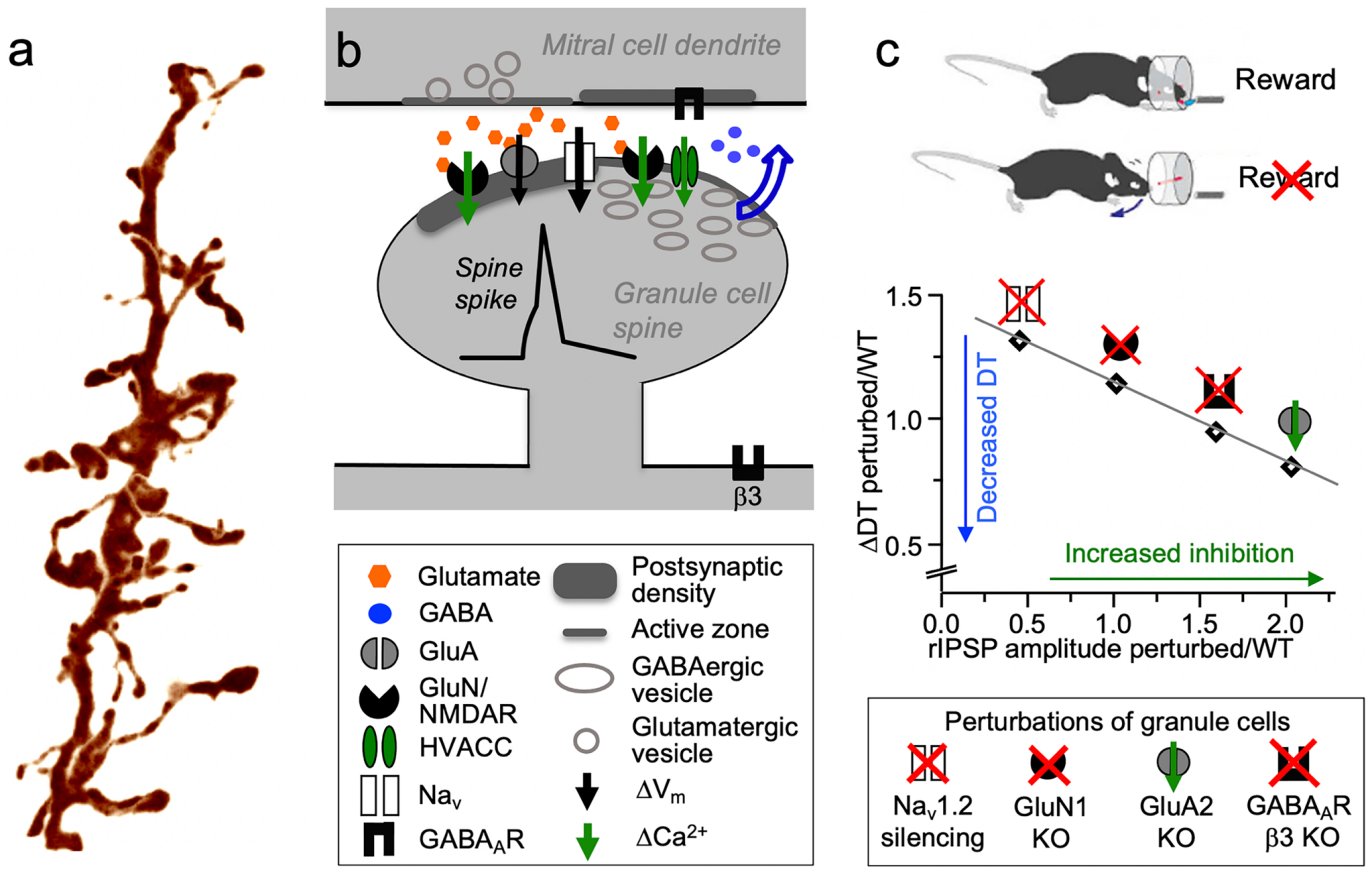
Granule cell morphology, synaptic signalling at the reciprocal spine and behavioral effects of GC manipulation. **a** 3D projection of an adult mouse GC dendrite segment containing multiple gemmules with long and thin necks and large heads. Height of the image corresponds to 30 µm. Image taken by Daniel Nunes **b** Signalling cascade within the reciprocal spine (mod. from Lage-Rupprecht et al. 2020). Upon release of glutamate from the mitral cell dendrite, AMPARs (GluA) get activated, leading to depolarization of the spine, then activation of Na_V_s and NMDARs in the glutamatergic GC postsynaptic density and GABAergic GC active zone. The Na_V_s in turn drive further depolarization, i.e., the spine spike, which both activates HVACCs and enhances Ca^2+^ entry via NMDARs by additional relief of their Mg^2+^ block. HVACC- and presynaptic NMDAR–mediated Ca^2+^ currents cooperate to promote recurrent release of GABA onto the mitral cell dendrite **c** Top: behavioral go/no go paradigm for odor discrimination (mod. from Abraham et al. 2004). Middle: Plot of discrimination time difference (∆DT) ratio versus recurrent inhibition ratio determined in gene deletion versus control mice (Abraham et al. 2010, Nunes and Kuner 2015, 2018). ∆DT is the difference in DT between monomolecular and binary mixture discrimination, reflecting the additional time needed to process the more similar stimulus. Linear regression *r*^2^ = 0.96, *F* = 43, *p* = .02

Aside from presynaptic inhibition of olfactory receptor neuron terminals, there are two main forms of inhibition of the principal neurons: (1) recurrent inhibition, which refers to interneuron feedback onto an exciting MTC, and (2) lateral inhibition, which refers to interneuron feedforward onto MTCs other than the exciting MTC. GCs, the most numerous neuron population of the OB, participate in both recurrent and lateral inhibition of MTCs at the level of the EPL, where they receive glutamatergic synaptic input from MTC lateral dendrites which they provide with reciprocal GABAergic output. Axonless GCs feature basal dendrites and an apical dendrite, both spinous, with the apical dendrite extending from the granule cell layer (GCL) into the EPL and contacting MTC lateral dendrites via large spines that house the reciprocal synapses (Fig. 1a). Historically, GC-mediated recurrent inhibition of MTCs has been suggested to provide substantial gain control for the massively convergent excitatory input onto MTCs, and GC-mediated lateral inhibition has been proposed to play a key role in contrast enhancement analogous to the visual system (Yokoi et al. 1995; Shepherd et al. 2007). However, a general chemotopic, continuous representation of molecular receptive ranges (i.e., map) could not be confirmed in the OB (e.g., Murthy 2011). Nevertheless, non-topographical representations may also use lateral inhibition for decorrelation of similar odorant representations (Cleland and Sethupathy 2006). Both the nature of olfactory mapping and the specific role of GCs in it are still unclear, in part due to several enigmatic or confounding observations.

## Enigmatic features of granule cells

### Purpose of the reciprocal spine as a unique circuit motif

Dendrodendritic, reciprocal excitatory-inhibitory synapses are rare on a brain-wide scale, but common to occur between principal neurons and local interneurons of the olfactory bulb (Crespo et al. 2013). Here, they are mostly formed not only between MTCs and GCs but also between MTCs and other inhibitory neurons of the glomerular and external plexiform layers. Uniquely, the MTC-GC reciprocal synapse forms on spines that consist of a rather large spine head connected by a thin elongated neck to the GC dendrite, a structure also referred to as gemmule (Fig. 1a, b) (Rall et al. 1966). The resulting dedicated anatomical compartment could enable an isolated reciprocal microcircuit. This observation has led us to propose the idea of the spine as a mini-neuron that can release GABA independently from the level of activity of its mother GC (Egger and Urban 2006). However, similar, functionally independent local processors are known to also exist in smooth dendrites (e.g., A17 amacrine cells of the retina (Egger and Diamond 2020)). What then is functionally particular about the gemmule architecture?

### Mechanisms for recurrent GABA release

Early in vitro studies of recurrent inhibition in individual MTCs delivered contradictory results based on using very strong stimulation paradigms in the absence of extracellular Mg^2+^: the extended release of large amounts of glutamate from MTC dendrites caused massive Ca^2+^ entry and slow IPSCs lasting for seconds. Some groups identified NMDA receptors as the main effector of recurrent GABA release, whereas others implied high-voltage activated Ca^2+^ channels (HVACCs), but did not provide data for an involvement of voltage-gated Na^+^ (Na_V_) channels as in “classical” axonal release (Schoppa et al. 1998; Isaacson and Strowbridge 1998; Chen et al. 2000; Halabisky et al. 2000; Isaacson 2001). Moreover, the recurrent inhibition detected in these paradigms is not necessarily originating from GCs but might also be exerted by other EPL interneurons such as PV+ cells. PV+ cells, however, do not feature a substantial NMDAR current, and NMDA receptor antagonists are currently used to separate GC-mediated inhibition from that exerted by other glomerular interneurons and possibly also PV+ neurons (Najac et al. 2011; Geramita and Urban 2017). Hence, what exactly are the mechanisms of recurrent GABA release?

### Lack of proof for functional reciprocal connectivity from individual GCs onto MTCs

Despite the aforementioned functional demonstrations of recurrent inhibition and solid ultrastructural evidence for the reciprocity of most GC spines within the EPL, functional connectivity from individually identified GCs to MCs has not yet been observed: several groups including ourselves (e.g., Egger et al. 2003; Schoppa 2006a) have been unable to find connected GC → MC pairs in the first place and even paired recordings of coupled MC → GC pairs have never shown a reciprocal connection so far (Isaacson 2001; Kato et al. 2013; Pressler and Strowbridge 2017). While this issue might to some extent be explained by the short electrotonic length of the thick MC dendrite, the complete lack of such observations is surprising in view of the many recordings of recurrent IPSCs and high density of GABAergic synapses on the MC lateral dendrites, most of which indeed originate from GCs (Bartel et al. 2015; Sailor et al. 2016; Matsuno et al. 2017). Yet, does functional reciprocal connectivity exist?

### Sparse global GC spiking

Although axonless, GCs are capable of firing full-blown APs and at the same time feature a rather hyperpolarized resting membrane potential (Egger and Urban 2006). Indeed, early studies found sparse spontaneous spiking activity and low odor-evoked excitability in GCs in vivo under anesthesia (Wellis and Scott 1990; Cang and Isaacson 2003), yet higher activities were found in awake mice (Kato et al. 2012; Cazakoff et al. 2014, Wienisch and Murthy 2016). Still, spike rates appear considerably lower than those of MCs. Can global APs thus power substantial GC-mediated lateral inhibition? Moreover, it is unclear whether GC spiking is predominantly driven by MTC dendritic and/or axonal input or other excitatory input sources, such as centrifugal projections (Kishi et al. 1984; Schoppa 2006a; Pressler and Strowbridge 2017).

### Little inhibitory drive

In spite of the high density of reciprocal synapses, GCs are unlikely to provide substantial inhibitory drive, because their silencing had little effect on mitral cell spiking rate in vivo (Fukunaga et al. 2014). Rather, they influence the relative timing of MC spiking, thus reinforcing older findings on a role in the generation of gamma oscillations at the reciprocal synapses (reviewed in Kay 2015). Other interneuron subtypes, both in the glomerular layer and in the EPL, are by now known to fill this gap, allowing for gain control of the highly convergent excitatory input onto MTCs. In the EPL, the aforementioned PV+ neurons have been shown to provide efficient and broad, long-range lateral inhibition between MTCs via reciprocal interactions with their large smooth dendritic arbors that extends horizontally within the EPL. PV+ neurons are broadly tuned for odors and suppression of their activity in vivo results in a general increase in MTC activity (Kato et al. 2013; Miyamichi et al. 2013). Do GC finally make only a minor contribution to recurrent MTC inhibition?

### Apparently missing contribution to lateral inhibition

Yet more strikingly, in the aforementioned in vivo recordings from MCs by Fukunaga et al (2014), a subset of MCs responded exclusively with inhibition to odor activation, presumably via lateral inhibition—but optogenetic silencing specifically of GCs had no effect on this lateral inhibition both in anesthetized and in awake animals, whereas silencing of glomerular inhibitory neurons did. Thus, GCs dominate neither recurrent nor lateral inhibition in terms of magnitude, i.e., total charge transfer, as also supported by other recent studies (Geramita and Urban 2017). On these grounds, Burton (2017) has rightfully challenged the central role that GCs were thought to play in olfactory processing. What then is the function of GC-mediated inhibition? Does it exist at all?

### Reciprocal GC output relevant for odor discrimination behavior

In spite of the results described above, deletion of glutamate receptors in GC changed the time mice required to discriminate highly similar odors (Abraham et al. 2010). Because of the activation of substantial numbers of glomerular input channels and highly similar activation patterns, in particular, lateral inhibition would be expected to be crucial for efficient discrimination. Yet, owing to technical limitations, only recurrent inhibition could be assessed in glutamate receptor-deleted mice. Removal of the GluA2 subunit increased recurrent inhibition and accelerated discrimination time, while removal of the GluN1 subunit showed the opposite effect (Abraham et al. 2010). While the viral expression of Cre recombinase used in this study is OB layer-specific, but not cell type-specific, it can be ruled out that PV+ interneurons of the external plexiform layer mediated these effects, because they mostly express AMPA receptors lacking the GluA2 subunit and negligible amounts of NMDA receptors (Kato et al. 2013; Miyamichi et al. 2013). Thus, molecular GC perturbations have a behaviorally relevant effect. How does this align with the above mentioned enigmas?

In the following, we review own recent findings that offer a clue to resolve these issues.

## Recent findings 1: recurrent inhibition by the reciprocal spine and role of GC action potentials

Via high-resolution two-photon imaging and two-photon uncaging of glutamate, we have recently gathered evidence that upon unitary glutamatergic input the reciprocal spines fire local action potentials (“spine spikes”) that in turn activate HVACCs. This mechanism relies on the tight electrical isolation of the spine head by the high spine neck resistance, boosting the AMPAR-mediated EPSP (Bywalez et al. 2015). Next, we found that mouse GC spines express clusters of the Na_V_1.2 subunits, and by knocking down Na_V_1.2 in GCs, we showed that upon eliciting action potentials (AP) in MTCs, the recurrent rIPSPs were strongly reduced. These results demonstrated that an AP was required for GABA release at the reciprocal synapse, just like neurotransmitter release from regular axonal presynaptic terminals (Nunes and Kuner 2018). Finally, by recording from mitral cells while activating single reciprocal spines with two-photon uncaging, we have been able to detect recurrent GABA release with a low release probability of approximately P_r_GABA_ = 0.3. This GABA release was indeed dependent on the local spine spike and HVACC activation, consistent with the mini-neuron hypothesis (Lage-Rupprecht et al. 2020).

Interestingly, recurrent GABA release was also dependent on NMDAR activation, pointing towards a cooperative mechanism for which we obtained further evidence via ultrastructural demonstration of NMDARs within the GABAergic active zone of the spine (Fig. 1b; Lage-Rupprecht et al. 2020). This opens the possibility of cooperative overlapping Ca^2+^ domains between NMDARs and HVACCs within the GABAergic active zone and, in particular, may establish a close distance between NMDARs and the release sensor at the readily releasable vesicles. Both HVACC- and NMDAR-mediated Ca^2+^ currents may overlap if the spine spike causes a NMDAR-mediated Ca^2+^ spikelet via fast, transient additional relief of the Mg^2+^ block, as illustrated by our simulations (Aghvami et al. 2019; see Fig. 6 in Lage-Rupprecht et al. 2020). However, because of the very slow activation kinetics of NMDARs (12–13 ms (Monyer et al. 1992; Wyllie et al. 1998)), this mechanism will unfold its full potential if NMDARs are still activated from a previous episode of glutamate release. Yet even when newly activated, Ca^2+^ can enter also at this early phase, although at a reduced amount. This may explain the low release probability of 0.3 reported in Lage-Rupprecht et al. (2020).

In summary, a cooperative action of Ca^2+^ entering through HVACC and NMDAR will maximize the release probability for GABA at the active zone and hence provides a conditional mechanism for GABA release. Neither of the two Ca^2+^ sources can achieve this in isolation. Importantly, the spine AP is instrumental to boost Ca^2+^ entry through both NMDAR and HVACC: (1) a rapid relief of Mg^2+^ block (Kampa et al. 2004) already during the upstroke of the AP permits nearly instantaneous Ca^2+^ entry through already gated NMDARs, and (2) during the repolarization phase of the AP, the driving force for Ca^2+^ entry through HVACCs will strongly increase (“tail current”; see Nunes and Kuner 2018) while NMDARs are getting blocked again by Mg^2+^.

An important consequence of this cooperativity is that there will be only little AP-mediated release from a reciprocal spine without concurrent synaptic input onto this spine. As to the generation and spread of APs within the axonless granule cells, the preferred site for spike generation is likely to be located on the proximal apical dendrite (Pressler and Strowbridge 2019), consistent with the strong difference in Na_v_1.2 channel density between the soma and the proximal apical dendrite of the GC (Nunes and Kuner 2018). Within the dendritic domain, NaV1.2 is rather homogenously distributed, yet forming local clusters (Nunes and Kuner 2018). As argued by this study, a certain density of Na_v_ channels within the clusters may facilitate AP generation. An AP will propagate along the GC apical dendrite and into its reciprocal spines perfectly well, as demonstrated by Ca^2+^ imaging and Na_v_ channel blockade, notwithstanding the electrical isolation from spine head to the dendrite (Egger et al. 2003; Pressler and Strowbridge 2019). The ensuing spine depolarization V_m_(t)_SPINE_ will closely resemble that of the spine spike (see also Aghvami et al. 2019), which on its own, in the absence of glutamate at the spine, has only a low probability to trigger GABA release, as we have argued above. Therefore, any AP that propagates within the GC has a low probability of causing lateral inhibition by releasing GABA. Only those spines containing NMDARs activated within a time window of a few milliseconds to a few hundreds of milliseconds (depending on the NMDAR subtype (Monyer et al. 1992; Wyllie et al. 1998)), preceding the AP of the GC, will release GABA.

Taken together, the NMDAR provides a short-term memory of activity present in the respective MTC in a time window of a few ms to a few hundred milliseconds. We predict that the cooperative action of NMDAR and HVACC at the GABAergic active zone then allows for GABA release in three modes, with increasing probability: (1) unitary release upon single MTC input; (2) the same spine was already activated within the NMDAR memory time window and the subsequent glutamate release generates recurrent inhibition as described above, e.g., during a theta burst of an MTC; (3) the GC fires an action potential that enters all spines (or a subset, see below), but GABA is only released from those spines that were activated during the NMDAR memory time window, thereby generating lateral inhibition. In essence, short MTC bursts will facilitate recurrent inhibition and lateral inhibition will mostly affect MTCs that are currently active or were active very recently.

Notably, extracellular stimulation of the GC layer can evoke MC inhibition (e.g., Chen et al. 2000; Egger et al. 2003; Arevian et al. 2008). Perhaps some small P_r_GABA_ from spiking GCs remains in the absence of glutamate, which is then amplified by the large number of stimulated GCs. Alternatively, the stimulation activates other, yet unknown inhibitory inputs to MCs, e.g., from deep short-axon cells or axons of EPL interneurons (Nagayama et al. 2014, Burton 2017). In any case, for weak sensory input, the functional setup of the reciprocal GC spines allows for segregated recurrent inhibitory interactions of a given GC with several glomerular columns. Because we observed that plastic changes in response to sniff-like stimulation are likely to also occur independently in each spine (Chatterjee et al. 2016), spine-specific and perhaps column-specific synaptic plasticity might exist.

## Recent findings 2: dendritic integration and coincidence detection on the level of the entire GC

For stronger sensory input, activity from many spines will become summated and result in regional and global signaling via Ca^2+^ spikes and Na^+^ spikes (Egger et al. 2003, 2005; Pinato and Midtgaard 2003; Zelles et al. 2006) that will then possibly translate into lateral inhibition. Recently, we have characterized the properties of dendritic integration in GCs, with special focus on the transition between local/purely reciprocal signaling and global spike signaling. To this end, we have established a holographic uncaging system that allows for simultaneous multi-site stimulation, thereby preventing the inactivation of voltage-dependent conductances expected during sequential uncaging (Go et al. 2019). We find that even though GCs show a rather hyperpolarized membrane potential, a quite small number of coactivated spines on their apical dendrite is sufficient to trigger non-local, dendritic Ca^2+^ and Na^+^ spikes, and even global Na^+^ spikes require no more than ~ 9 simultaneous apical inputs (Mueller and Egger 2020). Thus, the threshold for lateral inhibition is low. Moreover, lateral inhibition may often occur in a restricted manner within a branch or small portion of the GC dendritic tree, due to the dendritic spikes that may not invade the entire apical dendrite or not propagate fully to the soma, as has also been observed in vivo (Wienisch and Murthy 2016, Wallace et al. 2017).

The coincidence of local input and global spiking within a spine substantially increased Ca^2+^ entry up to threefold, indicating that the low P_r_GABA_ observed upon unitary activation might be substantially enhanced by additional spike invasion. Even though the summation of Ca^2+^ signals is spike timing-dependent and might be even sublinear in the case of |∆t| < 10 ms, because of the local inactivation of voltage-dependent conductances (Na_V_s and Ca_V_s, ∆t: time interval between the local and global spike (Aghvami et al. 2019)).

## Recent findings 3: behavioral evidence for a central role for granule cell–mediated GABA release in olfactory processing

To assess the relevance of physiological and network mechanisms of OB computations, it is ultimately necessary to link these mechanisms to odor discrimination behavior. A commonly used behavioral test to investigate odor discrimination in rodents is the go/no-go task (e.g., Bodyak and Slotnick 1999), designed for mice to learn to associate an odor with a reward (Fig. 1c). After about 300–600 trials mice can discriminate even highly similar stimuli such as binary 60:40 versus 40:60 mixtures with accuracies exceeding 90% (Abraham et al. 2004). In addition to the learning curve and discrimination accuracy, the time required to discriminate two odors is a parameter tightly linked to the mechanisms of computations performed by the OB network and, hence, changes in synaptic processing and network function will have a high chance to affect odor discrimination time. The amount of time required by the OB network to discriminate two odors is governed by the similarity and strength of glomerular activity patterns and is independent of sampling behavior (Bhattacharjee et al. 2019). Hence, discrimination of two monomolecular odorants with different glomerular representations requires less time than discrimination of a binary mixture of these odors (Abraham et al. 2004). In a next step, odor discrimination time can be linked to recurrent inhibition, a parameter that can be readily determined by recordings from MTC.

As already discussed above, deletion of the GluA2 subunit from GCs, a manipulation that renders AMPA receptors Ca^2+^ permeable and strengthens recurrent inhibition, resulted in a gain of function phenotype accelerating discrimination time by 54 ms (Abraham et al. 2010). Conversely, deletion of the GluN1 subunit, causing a complete loss of NMDAR expression, decreased recurrent inhibition and slowed odor discrimination by 62 ms (Abraham et al. 2010). Addressing the role of Na_V_ channels in GABA release at the reciprocal synapse as discussed in detail above, Na_V_1.2 was identified as the sole Na_V_ channel alpha subunit expressed in mouse GCs (Nunes and Kuner 2018). Na_V_1.2 was found to be expressed in GC spines and knockdown of it prevented the GC from generating action potentials. Recurrent inhibition recorded in MTC was strongly reduced and discrimination of binary mixtures took 95 ms longer (Nunes and Kuner 2018). Finally, to probe the role of GABAergic inputs that modulate GC function, the GABA_A_ receptor β3 subunit was deleted from GCs, a perturbation that removes all GABAergic input to GCs (Nunes and Kuner 2015). This disinhibition of GC function increased recurrent inhibition and accelerated odor discrimination by 33 ms. When taking together these four molecular perturbations of GCs, performed over a time period of almost 10 years, the strength of recurrent inhibition perfectly well predicts the difference in discrimination time (Fig. 1c), demonstrating a simple generalizable relationship in olfactory bulb processing: more recurrent inhibition accelerates discrimination of similar stimuli and less recurrent inhibition slows it down. While these experiments only assessed recurrent inhibition, it is likely that also lateral inhibition scales according to this principle.

## Integration of findings into an emerging hypothesis on lateral inhibition

**Fig. 2:**
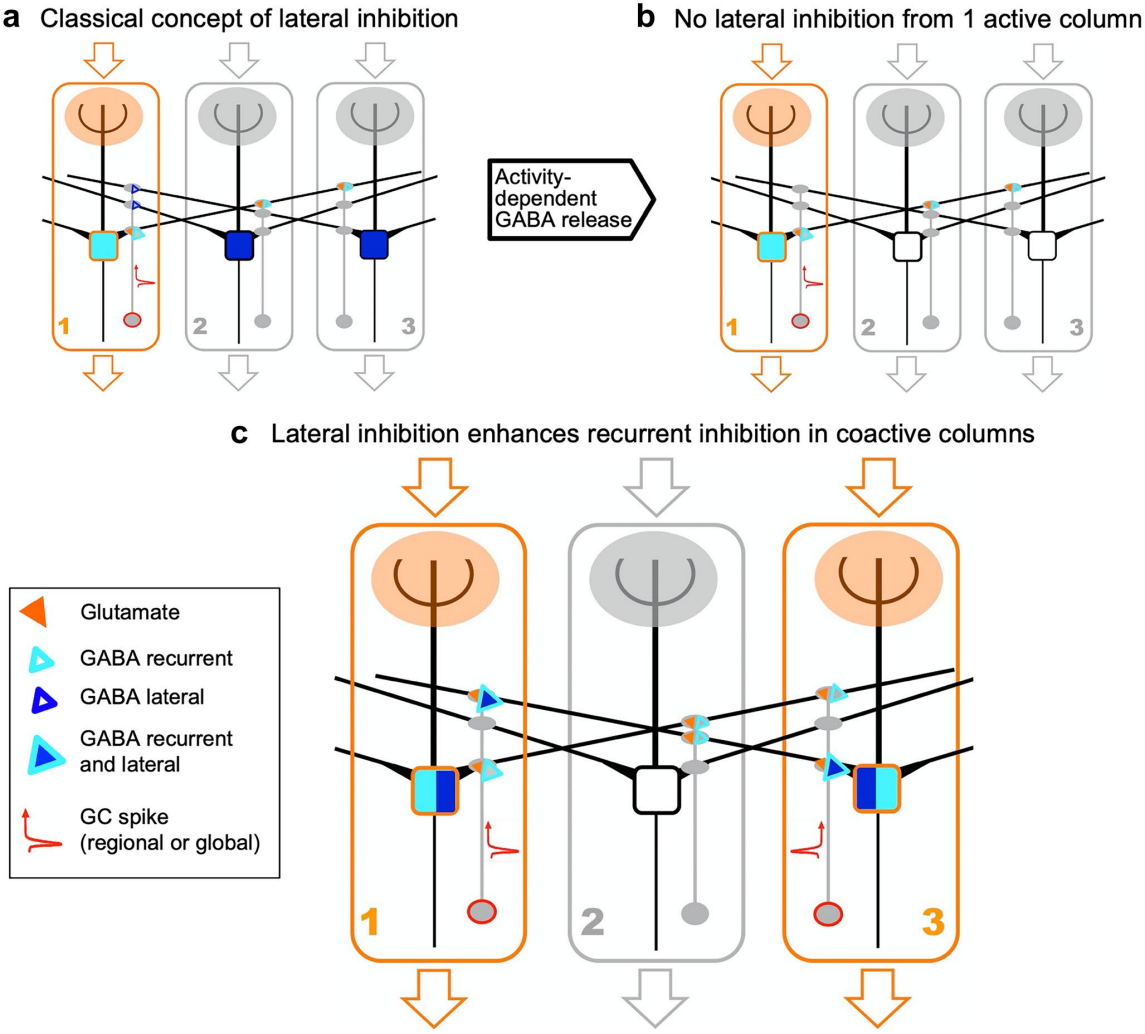
GC-mediated lateral inhibition: previous and new concept. In each schematic column, the depicted MC and GC stand for the ensemble of all MTCs and GCs belonging to the column. GCs are part of the ensemble if they can be fired by the active columnar MTCs. All three columns are interconnected via MC-GC-MC contacts. **a** Classical hypothesis. Column 1 gets activated by sensory input. Its MCs fire and receive recurrent inhibition from the GC spines they are connected to, both from within column 1 and from others. Upon firing of the columnar GCs, these release GABA from their reciprocal spines, including contacts to other columns and exert lateral inhibition there. **b** New hypothesis, scenario I: one active column. If only one column is activated, lateral inhibition will not happen since spines located in column 2 and 3 do not receive any glutamate and the propagating spike alone is not sufficient for GABA release. There will only be recurrent inhibition of column 1. **c** New hypothesis, scenario II: coactive columns. If two or more columns are coactive, the top spine in column 1 will exert recurrent inhibition of the coactive column 3, and this recurrent inhibition will be enhanced by the coincidence of the GC spike in column 1 with the lateral input; thus, lateral inhibition will also be exerted. If the connectivity and the strength of activation are symmetric, the situation in column 3 is also symmetric, resulting in mutual lateral inhibition and thus possibly synchronized output. Thus, the MCs within the active columns 1 and 3 will receive recurrent inhibition enhanced by lateral inhibition and the MCs in the silent column 2 will not be inhibited at all. Even if the GCs in column 2 would start firing, e.g., by multiple coincident lateral inputs, they will not release GABA on the MCs of column 2, since again, there is no glutamate at the respective contacts

Two main prerequisites are needed for lateral inhibition via GCs to occur:

(1) The involved GCs have to be excited sufficiently to generate a non-local spike (i.e., a spike that invades a larger dendritic compartment and its spines) or global spike. Evidence for global GC spiking upon uniglomerular activation in vitro has been observed by several groups, including our own (e.g., Schoppa et al. 1998; Egger 2008; Stroh et al. 2012; Burton and Urban 2015); as described above, rather low numbers of coactivated spines on GC apical dendrites (< 10) are sufficient to elicit global spiking and even less to elicit non-local, dendritic spiking (Mueller and Egger 2020). If a given GC can be fired via the activation of the MTCs associated with a particular glomerular column, it belongs to this column. Based on multiwire glomerular stimulation experiments (e.g., Chatterjee et al. 2016), we found that GCs can be fired from more than one glomerular channel independently (perhaps involving not only reciprocal synapses but also axonal MTC inputs). Thus, an individual GC may belong to more than one column, although the respective ensemble of columns will most likely be tightly spaced. In the classical picture (Fig. 2a), GABA release will happen not only recurrently at the reciprocal synapses that have received input from an active glomerulus, but also at other spines invaded by the spike that connect to MCs from other glomerular columns, exerting lateral inhibition. The spatial structure of this lateral inhibition depends on anatomical connectivity and network dynamics and has been proposed to be either isotropic, consistent with a center-surround field as in the retina or rather patchy (reviewed in Murthy 2011; see also Chae et al. 2019). Note, however, that most of the involved studies did not investigate specifically GC-mediated lateral inhibition. In any case, the columnar GC spines (i.e., those that receive synaptic input from the active column) will experience coincident local spine spikes and global spikes and therewith are likely to increase P_r_GABA_ at their GABA release sites.

(2) Moreover, because of the necessary activation of the NMDARs in the GABAergic active zone, there will be only little GC-mediated lateral inhibition upon activation of a single column because there is no glutamate provided at the lateral spines (Fig. 2b). Instead, we predict that GC-mediated lateral inhibition will happen specifically across coactive columns, such that a local synaptic input originating from a lateral active column will coincide with a global (or regional) columnar spike, resulting in enhanced inhibition of the lateral coactive column (Fig. 2c). The enhancing mechanism is the same as for the coincidence detection described above for recurrent inhibition within an active column. Provided that there are mutually interconnecting GCs in both columns, lateral inhibition will be symmetric. Interestingly, simulations have shown that columnar arrays of GCs may be particularly effective in mediating synchronization for symmetric lateral connectivity (McTavish et al. 2012), in line with our hypothesis.

Therefore, GC-mediated lateral inhibition acts non-topographically (i.e., not isotropically) to enhance recurrent inhibition. Non-topographical lateral interactions in the bulb have been proposed already previously by others based on functional evidence and simulations (e.g., Shepherd et al. 2007; Fantana et al. 2008; Urban and Arevian 2009). While non-topographical lateral inhibition may also happen within the glomerular layer via dedicated connections (e.g., Economo et al. 2016), GC-mediated non-topographical lateral inhibition does not require a specific, pre-established anatomical connectivity. Rather, it is a property of the outlined activity-dependent spine specificity of GABA release, by which functional lateral connectivity is dynamically restricted to coactive columns and thus matched to glomerular activation patterns.

This mechanism will allow GC-mediated lateral inhibition to participate in synthesizing the olfactory percept already at the level of the bulb from the individual glomerular columns, most likely via synchronization in the gamma band which is a widely accepted idea (e.g., Laurent et al. 1996; Kashiwadani et al. 1999; Schoppa 2006b; Brea et al. 2009; McTavish et al. 2012; Peace et al. 2017), while at the same time preventing unwanted energy expenditure and inhibition of other glomerular columns (i.e., if GABA would be released from all spines of an activated GC). Thus, inactive columns are not inhibited and therefore will remain sensitive for new stimuli or changing components.

In other words, the discovered mechanism allows GCs to perform lateral inhibition “on demand,” selectively on coactive mitral cells, and thus can provide directed, dynamically switched lateral inhibition in a sensory system with 1000 receptor channels, therefore explaining the unusual microcircuit of the GC spine/mini-neuron as a means of olfactory combinatorial coding.

Activity-dependent lateral inhibition was already observed earlier in vitro between pairs of mitral cells (MC) by Arevian et al. (2008); one “sending” cell was induced to fire at a high frequency, whereas the other “receiving” cell was either not activated at all or also subjected to evoked spike trains with increasing frequency. The receiving MC showed effects of lateral inhibition by the sending MC within a certain range of its own firing, but never for low or no own firing. The authors argue that the activity-dependent effect is due to increased recruitment of firing interconnected GCs with increased activity, because of the summation of inputs from both sending and receiving MC. While such summation can certainly contribute, our results further clarify the reason for a complete lack of lateral inhibition of silent receiving MCs.

Activity dependence is also reflected in the labeling of glomerular columns spanning all layers of the bulb by markers of neuronal activity (Kauer and Cinelli 1993). More recently, retrograde transsynaptic tracing studies using pseudorabies virus injections have further confirmed this concept, by labeling subsets of glomerular columns dispersed across the bulb, which very frequently contained a substantial fraction of GCs (Willhite et al. 2006; Kim et al. 2011). The authors already speculated that this patchy labeling could be explained by activity-dependent transsynaptic crossing from MC lateral dendrites, i.e., specifically to active GC spines, which matches with the powered release of GABA within activated columns as implied by the results discussed here.

## Summary: enigmas lifted

### Purpose of reciprocal spines as a unique circuit motif

The isolated spine is required to generate the localized spine spike via locally expressed Na_V_ channels and therewith the localized fast and cooperative activation of both HVACCs and presynaptic NMDARs situated in the GABAergic active zone. Thus, the design of the GC gemmule provides a local coincidence detection circuit, which is essential for OB function and at this point unique within the brain.

### Mechanisms for recurrent GABA release

The cooperation of HVACCs and NMDARs explains apparently contradictory early observations. Both require a local action potential to flux Ca^2+^ ions: HVACCs for activation and generation of the tail current and NMDARs for rapid relief of Mg^2+^ block. The interplay between NMDARs and HVACCs also could allow to render lateral inhibition conditional on activity within a time window of a few to hundreds of milliseconds in the connected MTC.

### Lack of proof for functional reciprocal connectivity from GCs to MTCs

The requirement of NMDAR activation explains why GC APs alone have a very low likelihood of triggering MTC inhibition. Hence, when testing functional connectivity by eliciting APs in the putative presynaptic cell, in this case the GC, the lack of connected MTCs is to be expected.

### Sparse global GC spiking

Most of the MTC-induced GC spiking activity will be limited to the gemmules (“mini-neurons”) of the apical dendrite or involve local dendritic spikes and will therefore not be visible to recordings from the GC soma. Furthermore, this spiking activity may also remain undetected in field potential recordings, because the limited space within which currents will flow might not suffice to generate sources and sinks far enough apart to be detected. In our view, global spikes mostly serve to enhance (but not trigger) a GC’s output across coactive columns.

### Little inhibitory drive

GCs do not prevent MC spiking but influence MC spiking frequency and therewith could contribute to the synchronization of coactive columns. In this scenario, recurrent inhibition is inherently linked to lateral inhibition.

### Apparently missing contribution to lateral inhibition

According to the model proposed here, lateral inhibition does exist, but predominantly between coactive columns and always in combination with recurrent inhibition, making it easy to miss experimentally.

### Reciprocal GC output relevant for odor discrimination behavior

Manipulations of essential components of the reciprocal microcircuit, i.e., deletion of the glutamate receptor subunits GluA2 or GluN1 and knockdown of the sodium channel Na_V_1.2 subunit in mouse GCs, consistently affected odor discrimination time, such that manipulations that both should reduce GABA release (Na_V_ KO, GluN1 KO) result in decreased recurrent inhibition and a slowed odor discrimination. Conversely, the GluA2 KO manipulation should increase GABA release (as well as the GABA_A_β3 manipulation that lowers the general inhibitory tone of GCs) and indeed increased recurrent inhibition and accelerated odor discrimination. Since according to our hypothesis lateral inhibition is tightly related to recurrent inhibition, it is likely to be affected in a similar manner. Hence, our framework is further closing the loop from molecules to synapses, networks, and behavior in olfactory bulb function.
